# Osteoarthritis, adipokines and the translational research potential in small animal patients

**DOI:** 10.3389/fvets.2024.1193702

**Published:** 2024-05-20

**Authors:** Lars F. H. Theyse, Emilia M. Mazur

**Affiliations:** Department for Small Animals, Soft Tissue and Orthopaedic Surgery Service, College of Veterinary Medicine, University of Leipzig, Leipzig, Germany

**Keywords:** adipokines, osteoarthritis, translational research, dogs and cats, joint homeostasis, joint disease

## Abstract

Osteoartritis (OA) is a debilitating disease affecting both humans and animals. In the early stages, OA is characterized by damage to the extracellular matrix (ECM) and apoptosis and depletion of chondrocytes. OA progression is characterized by hyaline cartilage loss, chondrophyte and osteophyte formation, thickening of the joint capsule and function loss in the later stages. As the regenerative potential of cartilage is very limited and osteoarthritic changes are irreversible, prevention of OA, modulation of existing osteoarthritic joint inflammation, reducing joint pain and supporting joint function are the only options. Progression of OA and pain may necessitate surgical intervention with joint replacement or arthrodesis as end-stage procedures. In human medicine, the role of adipokines in the development and progression of OA has received increasing interest. At present, the known adipokines include leptin, adiponectin, visfatin, resistin, progranulin, chemerin, lipocalin-2, vaspin, omentin-1 and nesfatin. Adipokines have been demonstrated to play a pivotal role in joint homeostasis by modulating anabolic and catabolic balance, autophagy, apoptosis and inflammatory responses. In small animals, in terms of dogs and cats, naturally occurring OA has been clearly demonstrated as a clinical problem. Similar to humans, the etiology of OA is multifactorial and has not been fully elucidated. Humans, dogs and cats share many joint related degenerative diseases leading to OA. In this review, joint homeostasis, OA, adipokines and the most common joint diseases in small animals leading to naturally occurring OA and their relation with adipokines are discussed. The purpose of this review is highlighting the translational potential of OA and adipokines research in small animal patients.

## 1 Introduction

Osteoartritis (OA) is a debilitating disease affecting both humans and animals. It affects diarthrodial or synovial joints which are essential for articulation between opposing part of the skeleton. Between two opposing long bones, the joint typically consist of two epiphyses covered with articular hyaline cartilage, connected by a joint capsule and integrated to form a single joint sac. This basic structure is present in all major joints, including the temporomandibular, zygapophyseal and sacroiliac joints. The articular hyaline cartilage consists of chondrocytes within an extracellular matrix (ECM) composed of collagen type II fibers and glycosaminoglycans (GAG). Glycosaminoglycans can be subdivided into 2 major constituents, including hyaluronic acid and aggrecan. Hyaluronic acid is an extremely long unbranched polysaccharide, whereas aggrecan consists of a protein core with numerous short unbranched sulphated mucopolysaccharides. These mucopolysaccharides consist of chondroitin 4-sulfate, chondroitin 6-sulfate and keratan sulfate. The aggrecan complex is connected to the hyaluronic acid by its linking proteins. Crosslinking between the collagen fibers and glycosaminoglycans is responsible for the rigidity of the cartilage matrix. The chondrocytes have a flat appearance near the joint surface and a more rounded appearance in the deeper layers of the cartilage. Chondrocytes are found as individual cells or as isogenic groups surrounded by a dense matrix known as capsule. The chondrocytes are responsible for the production and maintenance of the ECM. The collagen type II fibers in the ECM are anchored within the subchondral chondral bone. The subchondral bone lends stability to the overlying cartilage and thus to the joint surface. The subchondral bone has a direct vascular supply originating mainly from the blood vessels of the metaphyseal and epiphyseal system. Cartilage is almost completely depended on diffusion from the synovial fluid to cover its metabolic requirements. The synovia can be characterized as a plasma ultrafiltrate with a supplemental high amount of hyaluronic acid. The inside of the joint capsule is lined with synoviocytes consisting of type A and type B synovial cells. The type A or macrophagic cells predominantly function as scavenger cells. The type B or fibroblast-like cells are responsible for the production of hyaluronic acid and lubricin. Hyaluronic acid in the synovial fluid mainly acts as a lubricant during joint movement and forms an equilibrium with the hyaluronic acid in the ECM. Lubricin is a surface-active glycoprotein providing boundary lubrication and preventing cell and protein adhesion. As stated earlier the synovial fluid supplies all nutrients, oxygen and growth factors to maintain the chondrocytes and ECM. This means that there is a delicate anabolic and katabolic balance to maintain joint function (1). Joint autophagy plays an important role in this process as it allows the orderly degradation and recycling of cellular and ECM components within the joint (2, 3). Enhancement of joint autophagy has been described in early osteoarthritis (OA) (4). In human medicine, the role of adipokines in the development and progression of OA has received increasing interest.

The purpose of this review is to describe the mechanisms leading to OA, discuss the role of adipokines and explore the translational research potential in small animal dog and cat patients with osteoarthritic joint disease.

## 2 Osteoarthritis

Joints can withstand large loading forces, but show limited regeneration potential when damage does occur. With substantial damage to hyaline cartilage, a repair reaction will be initiated resulting in the formation of scar tissue cartilage. When the repair reaction is successful, the initial hyaline cartilage is replaced by fibrous cartilage. This fibrous cartilage is characterized by a depletion of chondrocytes and an ECM mainly containing collagen type I fibers with a coarse arrangement and only limited amounts of GAGs. The fibrous repair cartilage is clearly inferior in comparison with the initial hyaline cartilage. In many instances the repair reaction will not be successful with full thickness cartilage loss and subchondral bone eburnation as a result. The joint repair mechanism includes several additional responses related to the non-weightbearing bone surfaces within the joint sac, the joint capsule with the synovial membrane and the subchondral bone. The complete cascade of joint alterations, which can be partly considered to be a joint repair reaction, is known as OA. The terminology osteoarthritis emphasizes that there is a reaction from the bony structures and an inflammation of the joint. Any disturbance of the joint equilibrium can initiate an osteoarthritic response including chondrocyte apoptosis (5).

The classical division between primary OA and secondary OA has been supplemented with several subtypes including metabolic disease, mechanical disbalance and repetitive strain (6). In the concept of primary OA, the assumption is that there is no initial causative trauma to the joint and OA develops spontaneously and shows slow progression. In the concept of secondary OA, the assumption is that there is an initiating trauma with the development and progression of OA as a sequel. Problem with these concepts is that our understanding of OA and the available diagnostic tools have been and still are insufficient to assess and monitor OA at an early stage. The concept of early OA has been postulated to be able to detect OA before irreversible damage to the joint has been established. This would open up a window for regenerative intervention to stop progression of OA (7). Emerging concepts to assess the different stages and progression of OA are based on the description of clinical phenotypes and molecular endotypes (8, 9). Rational for these concepts is a targeted approach of OA treatment tailored to the disease stage. These concepts are still in development and clinical applicability is still limited. In the early stages, osteoarthritis is characterized by damage to the ECM and depletion of chondrocytes (10). Macroscopically, the cartilage surface will show fibrillation due to disruption of the ECM. Loss of ECM components into the synovial fluid will act as debris and initiate a response of the synovial membrane and in particular the type A synovial cells. The scavenging of debris by the type A synovial cells coincides with a clear inflammatory response of the synovial membrane. Pain receptors are mainly found within the synovial membrane and joint capsule. This explains the pain experienced during acute joint trauma and OA. The cascade of cytokine and adipokine driven inflammation will also exert its effect on the composition and amount of the synovial fluid produced by the type B synovial cells. These reactions initiate changes in the joint capsule. The synovial membrane shows villous hypertrophy and proliferation with a resulting enlargement of the synovial joint lining surface. Based on the function of the type A and type B synovial cells this could be beneficial by increasing the scavenging potential of the type A synovial cells and by increasing the synovial fluid production of the type B synovial cells. This mechanism is proposed to counteract ongoing joint damage and to re-establish and maintain joint homeostasis. Simultaneously, the joint capsule shows hypertrophy and proliferation of the fibrous capsule leading to a clinically noticeable thickening of the joint capsule. At the level of the non-weightbearing bone surfaces the repair response initiates a reaction where periosteum and joint capsule meet. The response starts with a proliferation of chondrocytes forming chondrophytes alongside the weightbearing joint surfaces. Interestingly, hyaline cartilage is formed and the process is similar to physiological endochondral ossification. In the next stage, ossification of the deeper cartilage layer is initiated forming osteophytes. Again, this progression of cartilage to bone is similar to physiological endochondral ossification. The resulting osteophytes are the typical radiographical feature of advanced OA. The combination of chondrophyte and osteophyte formation with thickening of the joint capsule leads to an overall enlargement of the joint. The chondrophyte and osteophyte formation within the joint can be considered to be an adaptive repair mechanism increasing the joint contact area in an effort to maintain joint function. The hypertrophy and proliferation of the joint capsule results in an increased stability of the joint with a concurrent loss of range of motion as a trade-off. Even with advanced osteoarthritic changes in a joint, individual patients can show remarkable good joint function with minimal clinical symptoms over a long period of time. As OA is progressive in nature, there will be a moment in time where equilibrium is lost and clinical symptoms will become noticeable. As osteoarthritic changes are irreversible, modulation of joint inflammation, reducing joint pain and supporting joint function are the only options. Drug treatment can alleviate clinical sign, but progression of OA can necessitate surgical intervention with joint replacement or arthrodesis as end-stage procedures.

## 3 The role of adipokines

Adipokines are cytokines produced by the adipose tissue that play functional roles in metabolic homeostasis, obesity and inflammation. Leptin was the first adipokine described (11). Since then adipose tissue has been regarded as an endocrine active tissue. In addition to adipose tissue, adipokines are expressed in many other tissues. In recent years, the role of adipokines in the development and progression of OA has received increasing interest (12, 13). At present, the known adipokines playing a role in the development and progression of OA include leptin, adiponectin, visfatin, resistin, progranulin, chemerin, lipocalin-2, vaspin, omentin-1 and nesfatin (14, 15).

Adipokines have been demonstrated to play a pivotal role in joint homeostasis by modulating anabolic and catabolic balance, autophagy, apoptosis and inflammatory responses (16, 17). In addition, the adipokines modulate subchondral bone turnover and chondrophyte and osteophyte formation. The interaction of adipokines and cytokines during the different phases of OA make it difficult to distinguish them as mainly inflammatory or anti-inflammatory. There are multiple signaling pathways by which the adipokines exert their effects and they are partly interdependent. This results in a complex network of action and effect which needs further elucidation (18). An overview of the reported main actions of the adopokines in respect to OA is presented in Table 1. Similar to humans, obesity is a major problem in domestic dogs and cats. Many of the deleterious effects of excessive adipose tissue deposition are proposed to be mediated through enhanced leptin production (19). In dogs, serum and synovial leptin concentrations correlated with joint pain and dysfunction (20) In many studies a direct correlation between the severity of OA and leptin plasma and synovia concentrations has been demonstrated (21–24) In contrast, several studies demonstrated a pro-inflammatory effect of adiponectin and resistin and a down-regulation of inflammation by leptin in patients with OA (25, 26). Leptin is considered to have a modulatory effect on the immune system resulting in the inflammatory characteristics of osteoarthritis and rheumatoid arthritis (27–29). In addition, leptin can induce a catabolic metabolism in chondrocytes, characterized by decreased autophagy and increased apoptosis (16, 30–33). Adiponectin has been described as a primarily anti-inflammatory adipokine (14). Nevertheless, adiponectin also demonstrates pro-inflammatory characteristics and suppression of chondrocyte metabolism in OA (12, 26, 34). Total adiponectin was significantly increased in women with advanced knee OA (35).

**Table 1:** Overview of adipokines in relation to the reported main action in osteoarthritis.

Adipokine	Main action in OA	Reference
Leptin	Pro-inflammatory	(13–33) (34–42),
Adiponectin	Anti-inflammatory	(12–15, 17, 18, 21, 24–26, 34–40)
Visvatin	Catabolic	(13–15, 17, 18, 21, 39)
Resistin	Catabolic	(13–15, 18, 21, 24, 25, 35, 38)
Progranulin	Anti-inflammatory	(14, 15)
Chemerin	Pro-inflammatory	(14, 15, 17)
Lipocalin-2	Immunomodulatory	(14, 15)
Vaspin	Immunomodulatory	(14, 15)
Omentin-1	Anti-inflammatory	(14, 15)
Nesfatin	Pro-inflammatory	(14, 15)

**Figure 1:**
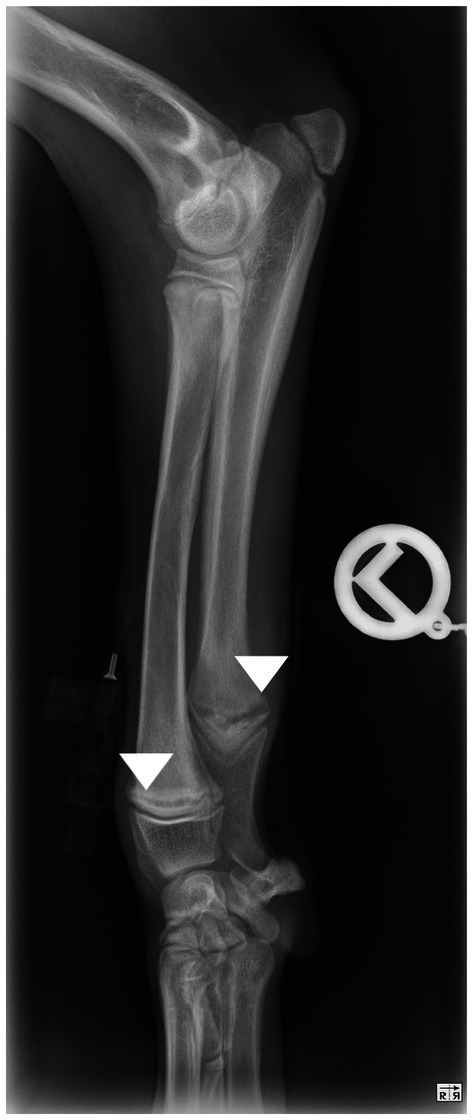
Mediolateral view of the antebrachium of a dog showing a translucent line parallel to the distal growth plates of radius and ulna typical for hypertrophic osteodystrophy (arrow heads).

The relation between OA and systemic and local concentrations of leptin and adiponectin remains contradictory and might represent a differentiation of adipokines expression in time (26, 36–38). In addition, local production of adiponectin and leptin has been demonstrated resulting in differences between local and systemic adipokines levels (39, 40). Information concerning the role of visfatin, resistin and other adipokines in OA is more limited (21, 39). As synovia is an ultrafiltrate of plasma, there is a direct link to endothelial vascular metabolism. Vascular endothelial growth factor (VEGF) plays a critical role in the homeostasis and regeneration of blood vessels including synovial membrane and subchondral vasculature. In view of this, the correlation between VEGF alterations and adipokines in synovial fluid and serum during OA is very interesting (41). As adipokines and VEGF exerts direct and indirect effects on bone metabolism and blood vessels, they might also be implicated in the development of several bone diseases including osteoporosis, osteochondrosis and osteochondritis dissecans (OC/OCD) like lesions, avascular necrosis of the femoral head and canine hypertrophic osteodystrophy (Figure 1) (42–45). In addition to clinical and animal model studies, *in vitro* studies using mechanical stress loading of osteoblast and chondrocytes and assessing their response to various adipokines might be instrumental in unraveling the complex interactions (13). This also allows for assessment of intracellular metabolism including mitochondrial function and endoplasmic reticulum stress (45–49). It is postulated that adipokines could play a causative role in the development of several joint diseases leading to OA. These naturally occurring joint diseases are discussed in the following section.

## 4 Animals as translational research models

### 4.1 The use of animal models

To study OA in humans, several animal models have been used (50). Most experimental studies have been performed using either mouse or rat models. In these short-lived species, OA does not occur spontaneously and has to be induced. The most common methods for surgical induction are destabilization of the medial meniscus with transection of the caudal meniscotibial ligament and destabilization of the stifle joint with transection of the anterior cruciate ligament (39). Destabilization of the stifle joint including damage of the joint cartilage can also be induced by intraarticular collagenase injection. The result is a rapid and aggressive development of osteoarthritis within several weeks. Disadvantage of these models is that they are markedly different from the natural development of OA (51). The use of knockout and transgenic mouse and rat strains with a genetic predisposition to develop OA better mimics the naturally occurring disease progress (2).

More suitable animal models would include species where the expected life span will be reached. In most commercially kept animals including cattle and swine this requirement is not met. Animals are either used for meat production, milk production or for reproductive means. Result is that the life duration is only a fraction of the expected life span. In contrast, small animal dogs and cats and horses usually are kept as pets or for sport where a commercially dictated limitation of life span is not an issue. Dogs and cats have a live expectancy of 10–16 years whereas this is 25–306 years in the horse. In all of these three species, naturally occurring OA has been clearly demonstrated as a clinical problem. Similar to humans, the etiology of OA is multifactorial and still nor fully understood (52). In the dog, skeletal maturity is reached at an age of 8 months. This means longitudinal growth has reached a plateau and growth plates close progressively. In the cat, the situation is similar to the dog with the exception of early age neutering before skeletal maturity is reached. Without sex hormones bony fusion of the growth plate is delayed rendering the growth plate vulnerable to trauma. In the horse, skeletal maturity is reached at 2 years of age. As OA is a clinical problem in dogs, cats and horses, they are potentially suitable as a naturally occurring OA patient models. In view of this, it is important to have an idea concerning the number of potentially available patients. In more detail, this would mean the number of dogs, cats and horses with access to veterinary medical care and potentially developing OA. Data on the numbers these animals kept as pets by owners in Europe and the United States of America are available. In Europe and the United States, felines haven the largest share out of all pets with 114 million and 70 million cats, respectively. The dog population in Europe accounts for 93 million animals with 90 million dogs in the United States. In comparison, the horse population in the United states and Europe is limited with 10 million and 7 million animals, respectively. Equine population numbers and patient size clearly pose limitations in considering the horse as a translational OA model. In this review, we will mainly focus on small animals. The high prevalence of OA in dogs and cats accounts for a comprehensive translational OA research potential.

### 4.2 Osteochondrosis and osteochondritis dissecans

**Figure 2:**
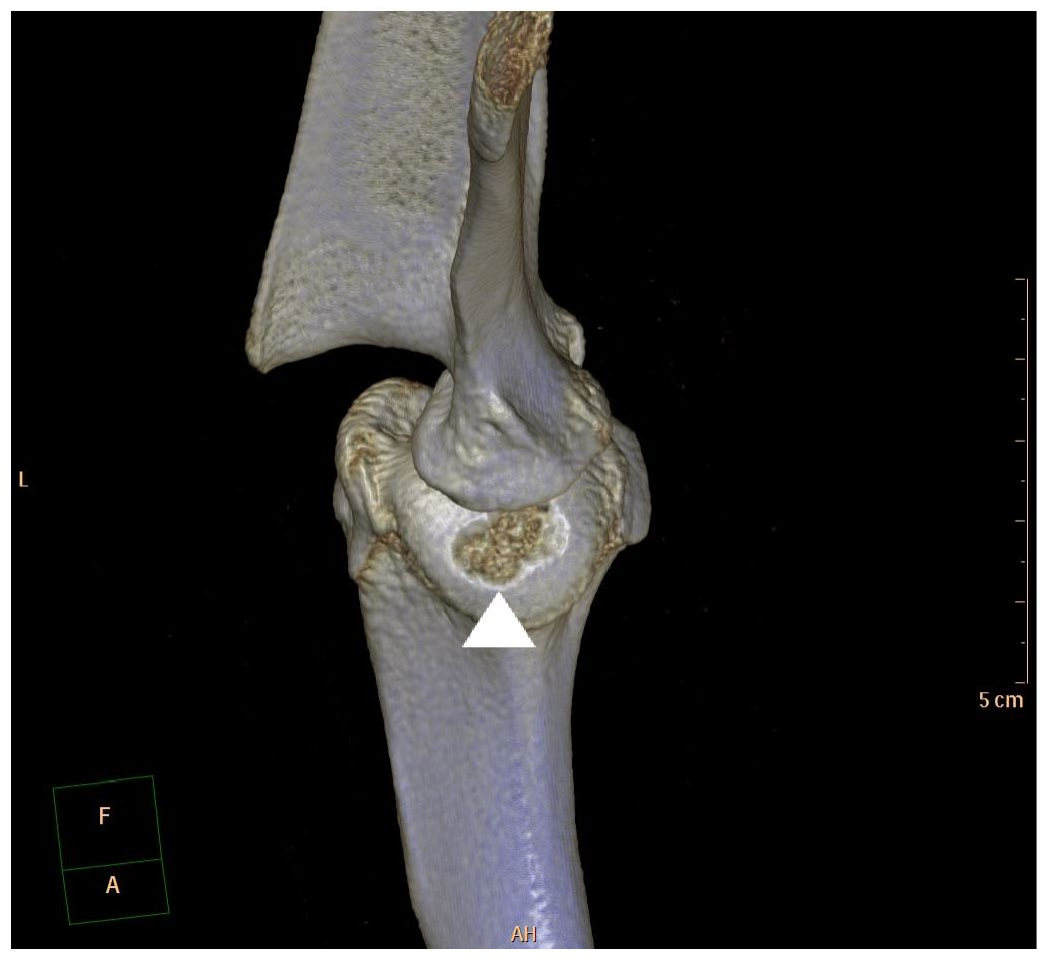
Caudocranial view of a 3D-CT reconstruction of a canine shoulder joint with a typical OCD lesion in the centrocaudal part of the humeral head (arrow head).

Like humans, actively growing dogs, cat and horses suffer from osteochondrosis (OC) and osteochondritis dissecans (OCD) (53). In these 3 species, a genetic susceptibility to OC/OCD has been demonstrated. Breeding interventions and dietary management have been able to decrease the prevalence of OC/OCD in dogs and horses without eradication the disease. The OC/OCD lesions are characterized by a disturbance in endochondral ossification. Initially, the focus of disturbance was thought to be within the cartilage with a delayed apoptosis of chondrocytes (54, 55). At present, vascular failure in the secondary ossification center of the epiphysis seems to be the key feature of OC/OCD (53, 56). The role of adipokines in this process is of great interest. Several joints can be affected and treatment in in analogy with the human counterpart. In dogs, the most common location is the shoulder joint and more specific the humeral head (Figure 2) although the disease also is a clinical problem in the elbow, crurotarsal joint, stifle, hip joint and lumbosacral junction (57, 58). In cats, OC/OCD has been described, but is far less important in comparison with horses and dogs (59). In the horse, the crurotarsal, stifle and metacarpophalangeal joints are the most common location for OC/OCD lesions. In OC lesions, where the disease is limited to the subchondral bone and the loadbearing cartilage joint surface is intact, healing can occur spontaneously. In these cases, the prognosis is excellent. In OCD lesions, where the loadbearing cartilage joint surface ruptures to form a cartilage flap ore even shows complete detachment with exposure of the osteonecrotic subchondral bone, clinical signs are obvious and surgical intervention is indicated. Even with proper surgical intervention, the OCD lesions usually are responsible for the onset of developing OA. As such, OCD lends itself as a naturally occurring translational patient model for first-to end-stage OA research.

### 4.3 Elbow dysplasia

In dogs, an additional important potential patient model for OA research is elbow dysplasia.

**Figure 3:**
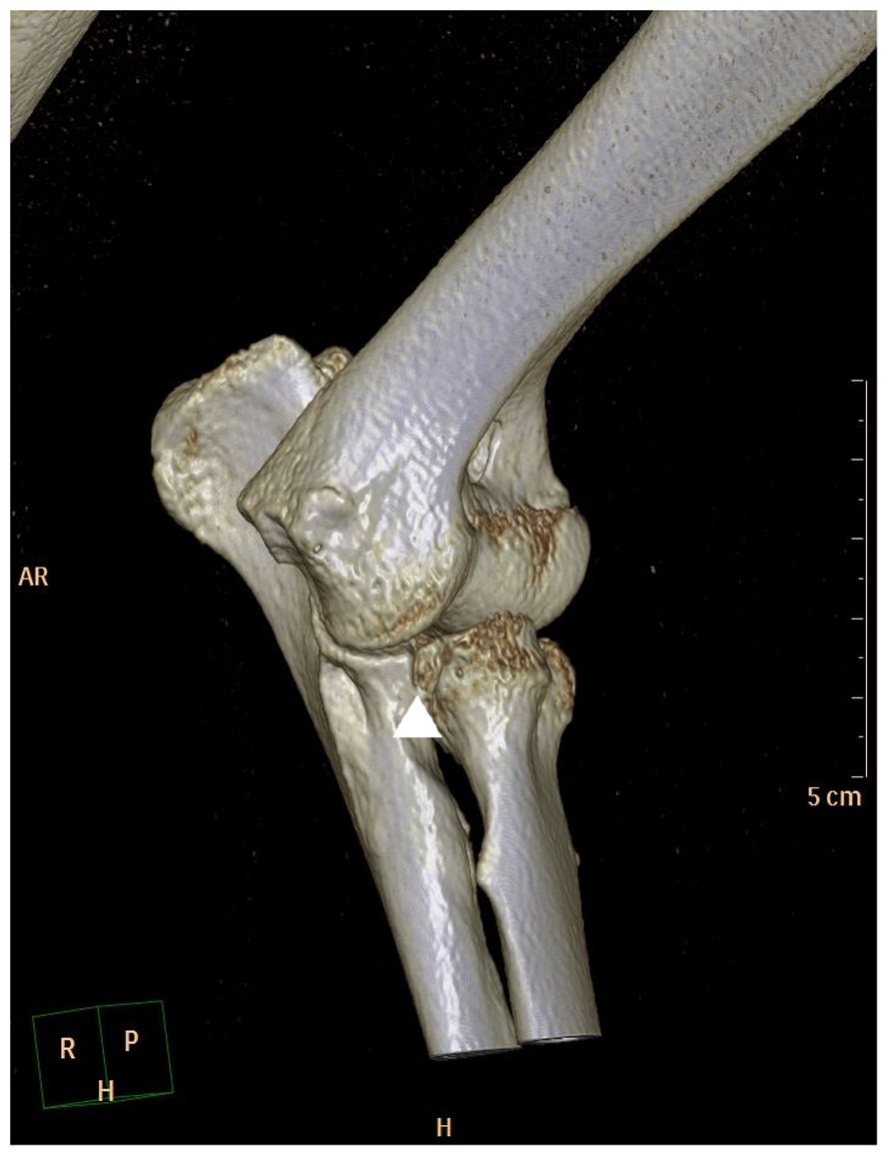
Craniomedial view of a 3D-CT reconstruction of a canine elbow joint with medial coronoid disease showing fragmentation of the coronoid and osteoarthritic changes (arrow head).

**Figure 4:**
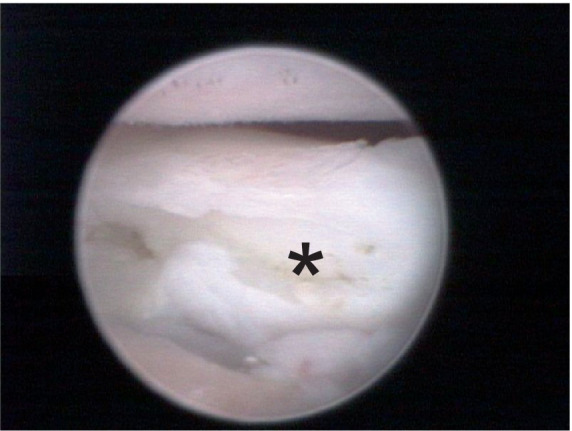
Arthroscopic medial view of a canine elbow joint with medial coronoid disease showing a displaced fragment with clear osteonecrosis (asterisk).

**Figure 5:**
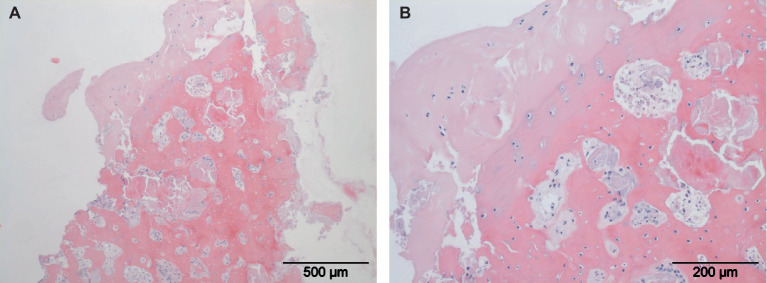
Histological slides of a typical osteochondral fragment. Local full thickness loss of articular hyaline cartilage is present **(A)**. Osteonecrosis with loss of osteocytes, cartilage degeneration and vasculitis are shown in larger magnification **(B)**.

Canine elbow dysplasia in the dog is a collection of partially independent and partially dependent diseases of the elbow joint (60). Elbow dysplasia consists of medial coronoid disease, elbow joint incongruity, ununited anconeal process, and also includes OC/OCD. The most important in numbers is medial coronoid disease. Medial coronoid disease shows similarities with OC/OCD (61). As is OC/OCD the lesion seems to originate in the subchondral bone of the medial coronoid process of the ulna in the direct vicinity of the radioulnar joint. In analogy with human anatomy, the radioulnar joint is the dog accommodates pronation and supination. For a dog the pronation and supination of the antebrachium is considered important to enable short turns and acute directional changes during locomotion. Medial coronoid disease is characterized by subchondral osteonecrosis, cartilage lesions and fragmentation of the apical part of the coronoid (Figure 3) (62, 63). The disease is considered to develop during the active growth phase prior to skeletal maturity which occurs at approximately 8 months of age. Clinical signs usually start around this time, but can also become noticeable later in life in conjunction with the presence of OA. At present, medial coronoid disease is the most important cause of front limb lameness in young dogs. The gold standard for treatment is arthroscopic partial coronoidectomy, including removal of osteochondral fragments and osteonecrotic bone (Figure 4) (64). The osteochondral fragments typically show osteonecrosis, cartilage degeneration and vasculitis (Figures 5A,B). Even with successful arthroscopic treatment, all dogs with medial coronoid disease will develop progressive OA (65). In specific cases, the disease can lead to the complete loss of all cartilage in the medial compartment of the elbow joint including both the medial part of the humeral condyle and medial coronoid. This condition is known as medial compartment disease. In all cases, medial compartment disease is accompanied by OA. Although medial coronoid disease has similarities with OC/OCD the etiopathogenesis of this joint disease is still unclear. Mechanical overload and joint incongruity have been postulated as potential factors. Several studies have shown a genetic predisposition with secondary environmental influencing factors including high energy diets, rapid growth rates and excessive exercise (66). The role of vasculitis as a primary factor leading to disturbance of osteogenesis during the process of endochondral ossification and secondary osteonecrosis has been postulated, but needs to be confirmed (63). The potential role of adipokines should be part of this research.

**Figure 6:**
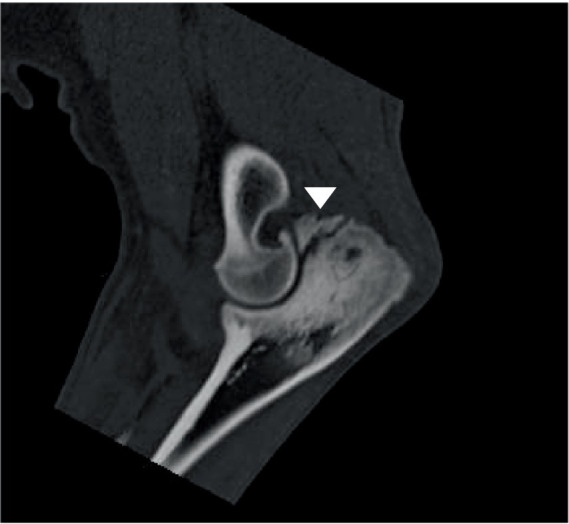
Sagittal CT view of a canine elbow joint showing a ununited anconeal process including severe osteosclerosis in the adjacent shaft of the ulna (arrow head).

In the dog, incongruity of the elbow joint is characterized by a step formation between the radial head and the ulnar medial and lateral coronoid process or malformation of the trochlear notch or radial notch. Incongruity has been described as a stand-alone disease or in conjunction with either medial coronoid disease or ununited anconeal process. A relative length deficit of the radius results in a radioulnar step formation with an abnormal weight distribution in the elbow joint with a potential overload of the medial and lateral coronoid of the ulna and a decreased load shearing by the radial head. This type of incongruity has been described in detail for the Bernese Mountain dog. Interestingly, medial coronoid disease and elbow incongruity have been shown to have a different genetic basis in this breed and can be transmitted independently (66). This also indicates that overload as a primary etiopathogenic factor in medial coronoid disease could be questionable. A relative length deficit of the ulna results in a radioulnar step formation with an increased load distribution on the anconeal process and radial head. This type of incongruity has been described in conjunction with an ununited anconeal process. An ununited anconeal process means a pathological separation between the anconeal process and the shaft of the ulna during the developmental growth phase of the proximal ulna (Figure 6) (60). As one of the types of elbow dysplasia, this disease shows similarities with OC/OCD. The ununited anconeal process shows the typical combination of vasculitis, osteonecrosis and osteosclerosis. As the trochlear notch and anconeal process are essential anatomical structures for elbow joint stability in quadrupeds, this disease results in joint instability, marked synovitis and rapidly progressing OA. Again, elbow incongruity based on an ulnar length deficit and united anconeal process can exist independently. To better assess the potential mechanisms at work in the canine elbow joint and antebrachium, it is important to understand the changes in function and appearance by breeding interventions on the wild type wolf as ancestor of the present domestic dog. Dogs have been bred for thousands of years for specific traits. These traits have been defined in the various pedigrees only in the last 100 years. These means that there is a vast range of body shapes and sizes ranging from an Irish Wolfhound weighing up to 70 kg (154 lb.) to a Chihuahua weighing less than 2,5 kg (5,5 lb.). Based on spontaneous mutation short-legged breeds have been used and bred as long as man and dogs have coexisted. These chondrodystrophic breeds show a shortening of all long bones with an equal distribution over the four legs. Problems typically can occur in the antebrachium as normal development relies on an even length development of the paired radius and ulna. As in humans, both the radius and ulna possess two growth plates with the critical exception that the radius has two epiphyseal growth plates and the ulna has one proximal apophyseal growth plate and one distal epiphyseal growth plate. This means that the development in length of the antebrachium between elbow joint and radiocarpal joint in relies on two epiphyseal growth plates in the radius and only one epiphyseal growth plate in the ulna. In the distal ulnar epiphyseal growth plate, the amount of endochondral osteogenesis and gain in bone length has to equal the osteogenesis und length development in the proximal and distal radial growth plates. To accomplish this the distal ulnar growth plate shows a functional growth plate surface to match the combined growth plate surface of both radial physes. This translates in an inverted cone shape of the distal ulnar growth plate typical for canines. This also means that the metabolic demand of this growth plate is particularly high resulting in a higher vulnerability for trauma and abnormal growth (67). During the development of the antebrachium there is a dynamic shift in the ulna from distal to proximal to accommodate the more equal length development in both radial growth plates. In chondrodystrophic breeds, this translates in a physiological antebrachial shortening with cranial bowing of the radius, a valgus originating in the distal antebrachium and minor antebrachial external torsion. This means that the elbow joint and antebrachiocarpal joint can function without limitation. Nevertheless, in comparison with normal-legged breeds chondrodystrophic dogs show a predisposition to develop elbow joint incongruity with a discrepancy in the degree of shortening of ulna in comparison with the radius. The result is a relative ulnar length deficit as discussed earlier. The tendency of these short-legged breeds which are typically also small in body size to develop an ununited anconeal process based on the incongruity is very small. As an exception, ununited anconeal process has been demonstrated in Bassett Hounds, a short-legged breed considerably larger in body size. In dog breeds with normal leg length, a predisposition for ununited anconeal process has been shown in the German shepherd dog (Figures 6).

In the dog, treatment of elbow joint incongruity is limited to the active growth phase as incongruity is accompanied by deformation of the humeral condyle, ulnar notch, radial head and radioulnar joint. As longs as the epiphyses are actively growing the joint has the potential to remodel. With the cessation of active growth at 8 month of age this adaptive mechanism is lost and the potential for amelioration reduced to a minimum. Nevertheless, elbow incongruity has been treated successfully, using circular external skeletal fixators and dynamic osteotomies (67). In case the ununited anconeal process is still in its OC-like stage, stabilization with an appositional or compression screw has been performed successfully in dogs. Spontaneous healing of the OC-like stage has also been observed. As soon as the ununited anconal process reached the OCD-like stage, removal of the dislodged anconeal process including local removal of osteonecrotic bone from the opposite ulnar shaft is indicated. As mentioned earlier loss of the anconeal process results in instability of the elbow joint and a rapid onset of OA.

In the cat, the range of motion of the antebrachium and radioulnar joint in pronation and supination is even greater and also plays a role in catching prey animals. In contrast to the dog, elbow dysplasia and in particular medial coronoid disease has not been described in the cat (59). Although spontaneous chondrodystrophy does occur in the cat, breeding with cats in order to capture this trait as a phenotype is prohibited as least in Europe. Nevertheless, OA of the elbow joint is very common in felines. Although OA in cats is receiving more and more attention, very little is known about the etiological factors.

During evolution, the horse has lost the potential of pronation and supination of the antebrachium with a functional fusion of the proximal ulna and radius. This means that in the horse the only range of motion is in flexion and extension. Similar to the situation in the cat, medial coronoid disease has not been shown to be a clinically relevant problem in the horse. In addition, elbow joint incongruity has not been reported as an orthopedic problem. Ununited anconeal process has been described in the horse, but with a very low prevalence (68).

### 4.4 Osteoarthritis of the carpal joint

The antebrachiocarpal, carpal and carpometacarpal joints is dogs and cats are very interesting when assessing their function. These joints are stable during loading in extension und show considerable laxity in relaxation. Their primary function under loading is to act as a shock absorbing structure while running and jumping. Whereas the joint angle is approximately 15 to 30 degrees in extension under normal weight bearing in a standing position, the extension can reach up to 90 degrees during vigorous exercise and jumps from a height. The stability of the antebrachiocarpal, carpal and carpometacarpal joint relies on the carpal bones and the short ligament interconnecting them and connecting them to radius, ulna and metacarpals. This configuration also exposes the carpus in dogs and cats to trauma. Repetitive or acute trauma is considered to be responsible for irreversible ligamentous carpal instability and OA in dogs and cats (69, 70). Carpal fractures and luxation have been described after major trauma and specifically in racing greyhounds (71). In dogs and cats, lameness due to carpal instability and OA is a common clinical finding. In racehorses, osteochondral chip fractures of the carpal bones have been reported. Repetitive or acute trauma during racing is considered to be the causative factor. Clinically interesting is the occurrence of carpal instability and OA in relatively inactive obese dogs and cats with a possible link to adipokines.

### 4.5 Hip dysplasia

Hip dysplasia is a very common disease in dogs, but also affects cats (72–74). In horses, hip dysplasia is not of clinical relevance. As in human patients, hip dysplasia is characterized by laxity of the hip joint with a tendency of the joint to subluxate (75). In this unstable situation, there is no normal loading contact between acetabulum and femoral head critical for the physiological development of the hip joint. The point load between the dorsal acetabular rim and femoral head will result in early onset joint cartilage damage and the onset of OA. In addition, the abnormal load distribution due to hip joint laxity will cause malformation and remodeling of the hip. In dogs and cats, this typically leads to a shallow acetabulum, incongruity between acetabulum and femoral head and finally progressive OA (76). Hip dysplasia typically affects young actively growing animals. Initially lameness is caused by the continuous instability during weight bearing and walking resulting in an acute and clearly painful synovitis (77). This initial phase of hip dysplasia lameness usually is present until 10 to 12 months of age. The hip joint tends to become more stable due to OA and remodeling of the capsule resulting in a decrease in lameness or even symptoms becoming subclinical. The second phase typically starts when the animals start suffering clinically from their secondary OA at an age of 3 years onwards. Obviously, there can be a clear overlap of the two phases. In severely affected dogs, lameness can be present continuously. For cats in general, due to their nature and behavior, it can be difficult to detect lameness. As in dogs, both hip joints usually are affected although the severity can differ from side to side. In case of bilateral disease this results in a symmetrical lameness. The only clinical signs in cats can then be a reluctance to jumps and showing reduced activity. This makes lameness assessment in cats a challenge even for skilled orthopedic surgeons. Hip dysplasia is a multifactorial genetic disease is dogs. The heritability rate is around 0.3 at best, meaning that environmental factors play a considerable role. Even with breeding interventions the prevalence of hip dysplasia in affected breeds has been at the same level for many years. Although hip dysplasia can affect any breed, it mainly is a disease of medium to large size dogs, including the Labrador and Golden retriever, Newfoundland, Rottweiler and German shepherd dog (75). In this context it is interesting to note that hip dysplasia is extremely rare in sight dogs, including greyhounds and whippets. These breeds have been genetically engineered for speed and visual hunting over hundreds of years. The sight dogs have long legs, a slender build with a small waist and a very deep thorax. In addition, the have very limited adipose tissue. Hip dysplasia is factually eradicated with this kind of phenotype. These observations would be consistent with a role of adipokines in the development of hip dysplasia. Treatment of hip dysplasia in dogs is symptomatic or surgical. In dogs under 1 year of age suffering from hip dysplasia, but without severe joint cartilage damage and without OA, a dual or triple pelvic osteotomy with outward rotation of the dorsal acetabular rim can be performed (78, 79). Aim of the procedure is to improve the dorsal coverage of the femoral head by the dorsal acetabular rim, a deeper seating in the acetabulum and to prevent subluxation. In cases where irreversible cartilage and progressive OA is already present a total hip replacement is the best option (80, 81). At present total hip replacement systems ranging from cats to giant breed dogs are available. Femoral head and neck resection can be used as a salvage procedure. In dogs and cats, pip dysplasia offers all possibilities to study the joint instability and the resulting OA. A relative disadvantage of the hip joint is that it is less accessible in comparison with the elbow joint, knee joint, carpus and tarsus.

### 4.6 Patellar luxation

Patellar luxation is a very common disease in small breed dogs and cats. The most common type in these animals is medial patellar luxation (82, 83). It is considered to be a developmental disease with a genetic predisposition in small animal (84). Patellar screening and breeding intervention have been used successfully to reduce the prevalence in certain breeds (85). Nevertheless, interventions have not succeeded in eradicating patellar luxation. Overweight during the active growth phase suggests an active role of adipokines in the typically small patients affected by patella luxation. Patellar luxation has been graded in veterinary orthopedics based on the severity of instability and the possibility to reduce the patella to within the patellar groove. The clinical classification is grade I when the patella can be manually luxated, repositions spontaneously while the animal shows no clinical signs. In grade II, the patella intermittently luxates with spontaneous reposition thus leading to intermittent clinical signs. In grade III, the patella is luxated permanently but can be reduced manually followed by immediate re-luxation. In grade IV, the patella is permanently luxated and manual repositioning is impossible. Animals with grade III and grade IV patellar luxation show continuous lameness with dysfunction of the quadriceps mechanism. The clinical signs are an inability to extend the knee joint to a normal weight bearing joint angle and an inward rotation of the lower leg. Especially animals with grade III and Grade IV show malformation of the knee joint. Due to the lack of physiological loading by the patella, the femoral trochlea does not develop properly and becomes shallow or even completely loses its groove. Patellar luxation in small animals is a very interesting model to study naturally occurring joint instability during the active growth phase. In addition, grade II patellar luxation is interesting as a chronic instability model. The deficiency of the quadriceps mechanism to stabilize the knee joint long-term results in degeneration of the cruciate ligaments. Treatment of medial patellar luxation grade II is typically based on a transposition of the tibial tuberosity in order to correct the malalignment of the quadriceps mechanism in combination with a reconstruction of the lateral femoropatellar ligaments and lateral joint capsule (86, 87). In grade II and IV, a trochleoplasty and corrective osteotomies may be necessary (83).

### 4.7 Cranial cruciate ligament disease

In dogs, cranial cruciate ligament disease (CrCLD) is the most important cause of hind limb lameness (88). In humans, anterior cruciate rupture usually is traumatic in origin and typically a sports injury. In contrast, canine CrCLD is degenerative in nature with the development of a slowly progressive deficiency of the cruciate ligament system. Clinically, heavily built obese dogs seem to be overrepresented, suggesting a link with adipokines. In conjunction, the cranial and caudal cruciate ligament form a single functional unit. Knee joint stability can only be guaranteed when both cranial and caudal cruciate ligaments are healthy. In dogs, both cranial and caudal cruciate ligaments are affected during CrCLD, but most of the developing instability can be attributed to the cranial cruciate. Degeneration of the cruciate systems leads to initial joint laxity, low-grade synovitis and early onset of OA (89). Symptoms can stay subclinical for an extended period. Acute clinical signs usually are related to sudden partial or complete rupture of the cranial cruciate with or without a lesion of the medial meniscus. At this stage, OA changes in the joint are already present. In view of the progressive degenerative changes in the joint and existing OA, cranial cruciate ligament replacement is not a viable option in dogs (90). Reconstruction of the anterior cruciate ligament with autologous tendons as in human athletes requires a joint without OA. This requirement is hardly ever met in canine patients. An additional problem following reconstructive surgery is that only limited loading of the joint is allowed initially. The duration of rehabilitation in humans is 8 to 12 months which is excessive for dogs considering their life expectancy. In dogs, the treatment of CrCLD is aimed at treating potential meniscal lesions and stabilization of the knee joint.

At present, the most popular surgical technique is the tibial plateau leveling osteotomy (TPLO). The leveling procedure brings the knee joint in a more flexed position, which results in a dynamic stabilization (91). Without weight bearing and active stabilization through the quadriceps mechanism, the knee joint still shows a positive drawer sign. The tibial leveling osteotomy does not address the cranial cruciate deficit in internal rotation. The end result is an improvement of knee joint stability without complete compensation of the cranial cruciate function. This means OA gradually progresses and secondary meniscal lesions can occur. Tibial tuberosity advancement (TTA) is another osteotomy technique (92).

By transposition and stabilization of the tibial tuberosity in a cranial direction, the direction of the patellar ligament is altered. The course of the patellar ligament is now parallel to the deficient or lost cranial cruciate. This results in a dynamic improvement of knee stability mainly based on the quadriceps mechanism. The TTA procedure is less effective in dogs with a very steep knee joint angle. Similar to the TPLO, the TTA procedure does not compensate for increased internal rotation of the tibia. Additional techniques used to treat canine CrCLD rely on intra-or extra-articular artificial ligament using bone anchors or bone tunnels with interference screws or buttons. These methods do not result in a true replacement of cranial cruciate function and do not address the degeneration of the caudal cruciate ligament. These techniques do have the potential decreasing internal rotation instability. Nevertheless, these artificial ligament techniques have similar limitations as the tibial osteotomy methods. A classic surgical treatment option to decrease cranial tibial translation and internal rotation in CrCLD is the lateral imbrication technique. This technique uses the lateral capsule of the knee joint to augment the patellar ligament and prevent internal tibial rotation. Although duration of rehabilitation is reported to be shorter after a TPLO procedure, the long-term result of all described technique is very similar. None of the techniques is able to prevent progression of OA. Progressive OA with ongoing thickening of the joint capsule gradually decreases joint instability and can even eliminate cranial drawer. The medial and lateral meniscus play a pivotal role to maintain knee joint stability. Lesion of the medial meniscus requiring partial meniscectomies due to CrCLD will have a severely destabilizing effect on the joint. In dogs with CrCLD where menisci remain intact, even conservative treatment can have a functional outcome albeit including OA. In summary, it should be clear that canine CrCLD will lead to loss of joint function and progressive OA in all cases (93, 94).

Traumatic cruciate and collateral ligament lesions most commonly in combination with meniscal tears occasionally occur in dogs, but are more common in cats. Ligamentous reconstruction, using artificial ligaments, bone anchors or bone tunnels with interference screws or buttons, is the surgical treatment of choice. In cats and small dogs, patient size can be a limiting factor.

### 4.8 Osteoarthritis of the tarsal joint

Tarsal OA is common in dogs and cats. As described earlier OCD of the talar bone in dogs leads to instability of the crurotarsal joint and the development of severe OA. Similar to the carpus, the tarsus acts as a shock absorber (95). The tarsus relies on its interconnecting ligament for stability. Repetitive ligamentous microtrauma is believed to results in joint instability with OA as a final result. Traumatic luxation of the crurotarsal, intratarsal and tarsometatarsal joint are common is dogs and cats (96, 97). Due to the complex anatomical configuration of the tarsus partial of complete arthrodesis is the treatment of choice (98, 99). In dogs, this usually is accomplished using screws and plate osteosynthesis. In cats, due to the size of the bony structures, external fixation systems are employed to reach partial or complete tarsal arthrodesis and regain stability. Intratarsal fractures are relatively common and a causative factor in tarsal OA (97, 100). Interesting is the occurrence of tarsal instability and OA in relatively inactive obese dogs and cats with a possible link to adipokines.

### 4.9 Osteoarthritis of the metacarpophalangeal and metatarsophalangeal joints

Dogs and cats have a foot typically consisting of 5 toes in the front limb and 4 toes in the hind limb. In analog with the human thumb, the first toe only has 2 phalanges. The first toe in dogs and cats is not weightbearing during locomotion. The functional weightbearing is attributed to the second and fifth toes and its metacarpals in the front and metatarsals in the rear. The second and fifth metacarpals and metatarsals and corresponding toes are important for lateral stability but less for weight bearing. These metacarpals and metatarsals are considerably shorter and smaller in size than the central third and fourth metacarpals and metatarsals. Most of the weight bearing load is transmitted through the central third and fourth metacarpals and metatarsals. The third metacarpal and metatarsals are slightly longer and larger in size than the fourth. In the horse, evolution has resulted in a third metacarpal and metatarsal, including its phalanges and hoof mechanism, taking all the load. Dogs and cats have complex metacarpal and metatarsal phalangeal joints. Each joint consists of the metacarpal or metatarsal with its corresponding first phalanx and three sesamoid bones. There is a paired sesamoid in the tendon of the interosseus muscles attaching caudoproximally on the first phalanx on the caudal joint surface. In addition, there is a small single sesamoid in the extensor mechanism on the cranial metacarpophalangeal and metatarsophalangeal junction. The caudal double sesamoids are crucial for joint stability and load absorption during weightbearing (101). In addition, the sesamoids protect the superficial and deep digital flexor tendons as they course over the caudal surface of the respective metacarpophalangeal and metatarsophalangeal joints. In front, dogs and cats have a large metacarpal pad protecting the metacarpophalangeal joint and each toe has its own digital pad. In the rear, the configuration is identical with a large metatarsal pad and four digital pads. During weightbearing, the load is distributed through the metacarpal, metatarsal and digital pads (102). This means dogs and cats literally walk on their toes with most of the load being transferred through the metacarpophalangeal and metatarsophalangeal joint. In view of this, it is no surprise that OA of the foot is an important cause of lameness in older dogs and cats. Although most of the weight bearing forces are transmitted through the central third and fourth metacarpo-and metatarsophalangeal joint, OA is typically more severe in the second and fifth toes. The reason for this distribution is presumed to be the increased exposure of this toes to traumatic shear forces (103). In the horse, all weight bearing forces are transmitted through the highly specialized third metacarpal and metatarsal bones and their corresponding metacarpophalangeal and metatarsophalangeal joints. This anatomical conformation is supposed to explain the development of OA in these joints. Interestingly, many dogs, cats and horses reach old age without developing OA in these specific joints. Again, adipokines are a likely factor predisposing for OA.

## 5 Conclusion

Humans, dogs and cats share many of the causative mechanisms leading to OA including overweight and obesity. There is a substantial number of dog and cat patients suffering from a variety of joint diseases leading to OA. With the steadily increasing demand for advanced veterinary medical care, the therapies for human and small animal patients are increasingly converging. With the present veterinary access to high-end diagnostic imaging modalities, including CT, MRI and arthroscopy and highly-developed surgical interventions, this opens up significant opportunities for collaboration between medical and veterinary physicians and researchers. In view of this, dog and cat patients can be considered as high-potential candidates for *in vitro* and *in vivo* translational OA and adipokines research without the obvious ethical restrictions. The great benefit would be a more reliable OA small animal patient model and a more focused and reduced use of laboratory animals.

## Author contributions

All authors listed have made a substantial, direct, and intellectual contribution to the work and approved it for publication.
